# GOAL ADJUSTMENT CAPACITIES DURING COVID-19: CONTEXT-DEPENDENT BENEFITS FOR EMOTIONAL WELL-BEING

**DOI:** 10.1093/geroni/igac059.2393

**Published:** 2022-12-20

**Authors:** Jeremy Hamm, Meaghan Barlow, Jaron Tan, Odalis Garcia, Katherine Duggan

**Affiliations:** North Dakota State University, Fargo, North Dakota, United States; Wilfrid Laurier University, Waterloo, Ontario, Canada; North Dakota State University, Fargo, North Dakota, United States; North Dakota State University, Fargo, North Dakota, United States; North Dakota State University, Fargo, North Dakota, United States

## Abstract

Increased constraints and lost opportunities inherent in the COVID-19 pandemic can threaten important life goals and erode emotional well-being. Theories of lifespan development have identified goal adjustment capacities (goal disengagement and goal reengagement) as core self-regulatory resources that can buffer against declines in well-being. However, little is known about the pandemic-related contextual circumstances under which goal adjustment capacities may become more or less beneficial for well-being. Using longitudinal data from a nationally-representative sample of Americans across the adult lifespan (aged 18-80, n=286), we examined the consequences of goal adjustment capacities for emotional well-being under circumstances when individuals reported lower or higher constraints than normal in their lives. Specifically, multilevel models tested whether the influence of between-person differences in (Level 2) goal disengagement and goal reengagement on well-being were moderated by (Level 1) within-person fluctuations in perceived constraints. Analyses controlled for age, sex, education, and income. We observed cross-level Goal Reengagement x Perceived Constraints interactions for depressive symptoms, perceived stress, and positive affect (bs = -.11 to .07, ps < .05), but not negative affect. Results showed that the benefits of goal reengagement for depressive symptoms, perceived stress, and positive affect were pronounced on occasions when participants reported lower (vs. higher) than average perceived constraints in their lives. Findings point to the moderating role of pandemic-related contextual circumstances and suggest that goal reengagement may be most beneficial when individuals have fewer constraints than usual in their lives and may thus able to capitalize on opportunities to pursue new attainable goals.

